# Genome Sequence of a Novel H14N7 Subtype Influenza A Virus Isolated from a Blue-Winged Teal (*Anas discors*) Harvested in Texas, USA 

**DOI:** 10.1128/genomeA.00520-16

**Published:** 2016-06-09

**Authors:** Andrew M. Ramey, Andrew B. Reeves, Rebecca L. Poulson, Deborah L. Carter, Nicholas Davis-Fields, David E. Stallknecht

**Affiliations:** a U.S. Geological Survey, Alaska Science Center, Anchorage, Alaska, USA; b Southeastern Cooperative Wildlife Disease Study, Department of Population Health, College of Veterinary Medicine, The University of Georgia, Athens, Georgia, USA

## Abstract

We report here the complete genome sequence of a novel H14N7 subtype influenza A virus (IAV) isolated from a blue-winged teal (*Anas discors*) harvested in Texas, USA. The genomic characteristics of this IAV strain with a previously undetected subtype combination suggest recent viral evolution within the New World wild-bird IAV reservoir.

## GENOME ANNOUNCEMENT

H14 hemagglutinin (HA) subtype influenza A viruses (IAVs) were first identified in the former Soviet Union in 1982 (1) and not detected again until 2010, when they were identified in samples collected from sea ducks sampled in the United States (2). Subsequently, H14 subtype IAVs have been identified in the United States and Guatemala (3–6). Previously, H14 subtype IAVs have been associated with neuraminidase (NA) genes of the N2 to N6 and N8 subtypes (2–7). While the identification of an H14N7 IAV is described here, H14 has not been detected in combination with the N1 and N9 NA subtypes.

As part of ongoing research and surveillance, we isolated an H14N7 virus from a combined oropharyngeal/cloacal swab sample collected from a blue-winged teal (*Anas discors*) harvested in Texas, USA (29°41′34.80″N, 94°37′44.40″W), on 20 September 2015. Given the detection of a previously unreported IAV subtype combination, we genomically sequenced this isolate and compared the data to information reported on the GenBank public database (as of 4 April 2016).

The deduced amino acid motif for the fusion cleavage site of the H14 HA gene segment of A/blue-winged teal/Texas/UGAI15-6890/2015 (H14N7) was PDKQTK, consistent with low pathogenicity in poultry and identical to all other H14 subtype IAVs identified in New World waterfowl. Sequence data for the H14 HA gene segment of A/blue-winged teal/Texas/UGAI15-6890/2015 (H14N7) shared ≥95% nucleotide identity with all other IAVs of this subtype identified in the United States and Guatemala, while the shared identity with H14 viruses from the former Soviet Union was lower (88%). The N7 NA gene segment sequence of A/blue-winged teal/Texas/UGAI15-6890/2015 (H14N7) virus shared 97 to 99% identity with BLAST hits for IAVs isolated exclusively from wild-bird samples collected within the United States.

The top BLAST results for internal gene segments of the A/blue-winged teal/Texas/UGAI15-6890/2015 (H14N7) virus included almost entirely IAVs isolated from samples collected within North America or Central America, with a few exceptions. These exceptions included two polymerase basic two (PB2) gene segment sequences for viruses isolated from thick-billed murres (*Uria lomvia*) in Greenland that shared 98% identity with A/blue-winged teal/Texas/UGAI15-6890/2015 (H14N7) and a single sequence for the nonstructural (NS) gene segment of A/gull/Peru/PuV172/2009 (H1N1) that shared 99% identity with the NS gene segment of this novel H14N7 IAV strain.

Comparisons among gene segment sequences for A/blue-winged teal/Texas/UGAI15-6890/2015 (H14N7) and those reported on the GenBank public database revealed that the vast majority of sequences sharing high identity (i.e., 95%) originated from North American and Central American avian-origin samples. These findings are suggestive of recent viral evolution (antigenic shift and drift) resulting in the emergence of A/blue-winged teal/Texas/UGAI15-6890/2015 (H14N7) within the New World wild-bird IAV reservoir. However, phylogenetic analyses are needed to confirm or refute this hypothesis. As with other IAV HA subtypes, H14 appears to form genomic constellations with numerous NA subtypes via reassortment. Additional sampling for H14 IAVs and genomic characterization of resulting isolates would be useful for evaluating the relative abundance of H14 HA and N1 to N9 NA subtype combinations in the wild-bird reservoir.

### Nucleotide sequence accession numbers.

The GenBank accession numbers for the complete sequence of A/blue-winged teal/Texas/UGAI15-6890/2015 (H14N7) are KX024580 to KX024587.
